# DeepBindRG: a deep learning based method for estimating effective protein–ligand affinity

**DOI:** 10.7717/peerj.7362

**Published:** 2019-07-25

**Authors:** Haiping Zhang, Linbu Liao, Konda Mani Saravanan, Peng Yin, Yanjie Wei

**Affiliations:** Joint Engineering Research Center for Health Big Data Intelligent Analysis Technology, Shenzhen Institutes of Advanced Technology, Chinese Academy of Sciences, Shenzhen, Guangdong, China

**Keywords:** Protein–ligand binding affinity, ResNet, Deep neural network, Native-like protein–ligand complex, Drug design

## Abstract

Proteins interact with small molecules to modulate several important cellular functions. Many acute diseases were cured by small molecule binding in the active site of protein either by inhibition or activation. Currently, there are several docking programs to estimate the binding position and the binding orientation of protein–ligand complex. Many scoring functions were developed to estimate the binding strength and predict the effective protein–ligand binding. While the accuracy of current scoring function is limited by several aspects, the solvent effect, entropy effect, and multibody effect are largely ignored in traditional machine learning methods. In this paper, we proposed a new deep neural network-based model named DeepBindRG to predict the binding affinity of protein–ligand complex, which learns all the effects, binding mode, and specificity implicitly by learning protein–ligand interface contact information from a large protein–ligand dataset. During the initial data processing step, the critical interface information was preserved to make sure the input is suitable for the proposed deep learning model. While validating our model on three independent datasets, DeepBindRG achieves root mean squared error (RMSE) value of pKa (−logK_d_ or −logK_i_) about 1.6–1.8 and *R* value around 0.5–0.6, which is better than the autodock vina whose RMSE value is about 2.2–2.4 and *R* value is 0.42–0.57. We also explored the detailed reasons for the performance of DeepBindRG, especially for several failed cases by vina. Furthermore, DeepBindRG performed better for four challenging datasets from DUD.E database with no experimental protein–ligand complexes. The better performance of DeepBindRG than autodock vina in predicting protein–ligand binding affinity indicates that deep learning approach can greatly help with the drug discovery process. We also compare the performance of DeepBindRG with a 4D based deep learning method “pafnucy”, the advantage and limitation of both methods have provided clues for improving the deep learning based protein–ligand prediction model in the future.

## Introduction

Many complex diseases still prevailed due to lack of effective therapeutic drugs; for instance, many type of cancers, dengue viral disease, Human Immunodeficiency Virus, hypertension, diabetes, and Alzheimer’s disease (Iyengar, 2013; Zahreddine & Borden, 2013). As the mechanism and targets of these complex diseases gradually being explored, developing effective drugs to block the disease related pathway by protein–ligand interaction becomes possible (Copeland, Pompliano & Meek, 2006). In the post genomics era, although some novel therapeutic methods, such as immunotherapy, have tremendously progressed, small molecule drug design is still a dominant way to combat diseases (Anusuya et al., 2018). About 70% approved drugs in the DrugBank database belong to the small molecule category (Wishart et al., 2008). Currently, the drug development is a long-term and costly process, spending about billions of US dollars and taking several years to develop a single on-market drug (Politis et al., 2017). In order to solve the paradox of increasing requirement for new drug and low efficiency of drug development, many researches are focused on developing computational methods to aid the drug discovery (Heifetz et al., 2018).

Some molecular drugs exert their therapeutic effect usually by blocking or activating protein targets. Computational virtual screening by molecular docking of ligands against protein target is a widely used procedure to identify active drug like molecules (Chen, Li & Weng, 2003; Verdonk et al., 2003; De Vries et al., 2007; Jayaram et al., 2012; Paul & Gautham, 2016). The docking procedures consider various binding conformations by rotation and transition of the ligands. Further, the ligand flexibility also was taken into account in some docking softwares (Trott & Olson, 2010). Some commercial and academic free docking softwares also consider the flexibility of protein as well, but are computationally more expensive (Friesner et al., 2004; Zhao & Sanner, 2007). Docking score was often used to estimate the protein–ligand binding affinity. A typical scoring function is usually based on physical or knowledge based and it usually contains Van der Waals interaction term, electrostatic interaction term, hydrogen bond term, a highly approximate solvation term and surface contact area term, sometimes even approximate entropic term (Guo et al., 2004; Chaudhary, Naganathan & Gromiha, 2015).

In recent years, there is a trend of using machine learning to predict the binding affinity from structural data (Ragoza et al., 2017; Jiménez et al., 2018; Öztürk, Özgür & Ozkirimli, 2018) and it is reviewed in detail (Ain et al., 2015; Wójcikowski, Ballester & Siedlecki, 2017). Comparing to the simplified and fixed scoring function, arbitrary functions were used in machine learning models that are capable of transforming the input to the output label in the training process. The machine learning approach allows greater flexibility in selecting features compared to existing scoring functions. Traditional machine learning requires predefined features based on expert knowledge. There are many protein–ligand complex structure datasets available, some with experimental binding affinity value (Colwell, 2018). These data can be used for training, validation, and testing for the developed protein–ligand prediction model. Recently, deep learning has achieved impressive success in image recognition and language processing. Since deep learning can easily create binary or multi-class classifiers or regressions, it has been relatively and widely used by bioinformaticians (Min, Lee & Yoon, 2017).

Deep neural network contains many more layers and enables the model stronger in identifying more complicated patterns (LeCun, Bengio & Hinton, 2015). Convolutional neural networks (CNNs) are suitable for image recognition. The convolutional operations reduce the number of weights tremendously compared with the fully connected neural networks. The filters which share same weight can extract features automatically from the data. There are several famous CNN models, for example, ResNet, which is the winner of ImageNet Large Scale Visual Recognition Competition 2015 in image classification. The deep learning approach benefits largely from the computational power of graphics processing unit.

Besides of the model architecture and various parameter settings, how to represent the protein–ligand interface data is a critical problem (Du et al., 2016). In a very recent work (Stepniewska-Dziubinska et al., 2018), the authors developed a method “pafnucy” by using four-dimensional (4D) matrix to construct the input data and three-dimensional (3D) coordinate information as an extra dimension about atom property. In order to reduce the computational spending, such method includes only the protein region that is present around the ligand. Using 4D matrices to represent the protein–ligand information can be very effective in keeping the spatial and chemical information, which is critical to determine the binding affinity. Another advantage is that the 4D matrix format is quite suitable for CNN learning.

Considering the above facts, we proposed a native-like protein–ligand identification method by applying the ResNet CNN model with a two-dimensional (2D) binding interface related matrix as input. To estimate the native protein–ligand effectively, the interface information, such as atom pairs, atom type, and spatial information were kept appropriately for balancing the computational efficiency and accuracy. Instead of using 4D to include all the spatial information and atomic type, we use 2D map to simplify the information as a picture like format. ResNet allows much deeper layers to identify more complex feature that may contribute to the protein–ligand binding affinity. Based on the data processing and ResNet model, we built a regression model that can accurately predict the protein–ligand binding affinity. By comparing our method performance in diversified datasets with other methods including traditional docking scores and 4D based deep learning scoring method, we show the generalized advantage and limitation of the current protein–ligand affinity prediction method, and provide helpful clues to overcome those limitations for protein science community.

## Materials and Methods

### Dataset

The protein–ligand binding complex coordinates and binding strength data are retrieved from PDBbind database version 2018 (Liu et al., 2015). The PDBbind dataset is a comprehensive collection of high-quality protein–ligand complex structures along with experimentally determined binding affinity values. The ligands with rare occurring atoms, such as SE, SX, are excluded in our atom type list. We excluded redundant complexes present in the CASF-2013 (Li et al., 2018), CSAR_HiQ_NRC_set (Dunbar et al., 2013) and Astex Diverse Set (Hartshorn et al., 2007) leading to a total of 15,425 crystallized protein–ligand complexes. The data were then divided into 13,500 training set, 1,000 validation set, and 925 testing set and these datasets are non-redundant and independent.

Several familiar protein–ligand complex datasets were chosen as independent test sets, including the CASF-2013 set, CSAR_HiQ_NRC_set, and Astex Diverse Set. Each of the datasets contains 195, 343, and 74 protein–ligand complexes, respectively. These datasets are used for testing the model performance as independent sets, and can help in detecting generalization problems related to database specific artifacts. The structures in three external datasets were prepared and stored in the format as in the PDBbind database. The range of experimental binding affinity for each class has been provided in Table S1A. The maximum binding affinity of the training set is around 13, the test set is around 9 for group A and B and 13 for group C, and for the validation set, it is 11 for group A and B and 14 for group C, respectively.

### Preparation of protein–ligand complexes

In order to standardize the atom name and type in the PDB coordinate file, the Amber tool was used to convert the ligand into mol2 format, and the protein into PDB format (Case, 2018). Except for the B atom type, all other atom types in the ligand are taken from the generalized amber force field. Together with the B atom type, a total of 84 atom types were used for the ligand. The protein atom type is taken from the Amber99 force field, with 41 types. The atom types are listed in Table S1B. We use one hot representation to encode ligand type and protein atom type, respectively, resulting in an 84-dimension one hot representation for each ligand atom type, and 41-dimension one hot representation for each protein atom type. Further, we grouped ligands in the dataset into three types such as A, B, and C based on Log*P* value (The group A have Log*P* < −1, group B have −1 ≤ Log*P* < 1 and group C have Log*P* ≥ 1, respectively) for analyzing the performance of our model which is presented in Table S2.

**Figure 1:**
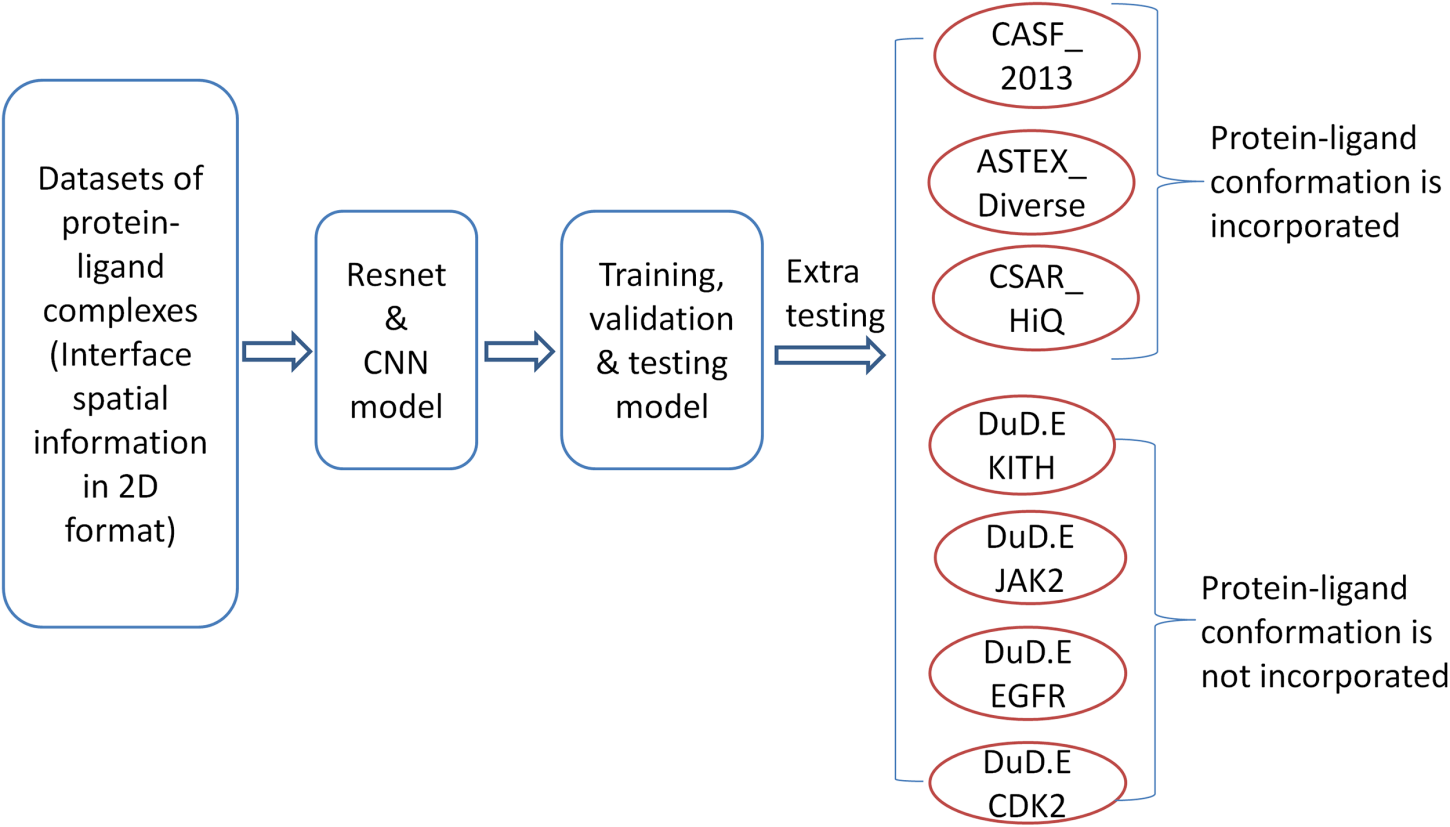
The workflow of model training and testing.

The workflow of the methodology is shown in Fig. 1. In the initial step, we consider a dataset from the PDBbind database and represent interface spatial information in 2D format. The data has been used to develop a predictor by CNN and the ResNet model. The resulting model is tested and validated by using standard procedures. Further, we validated our model on various independent datasets from DUD.E database with no native complexes. The detailed procedures for each and every step are presented in the following sections.

### Computation of protein–ligand atom pairs

We compute the interactions between protein atom and ligand atom in order to keep the critical contact information. Several cutoff values were tried, and we choose 0.4 nm as final setting. In order to keep the spatial information between the pairs, we cluster the protein atoms into five groups using kmeans from the sklearn package (Pedregosa et al., 2011). During the input file preparation, the atom pairs belonging to the same class group were written nearby. In this way, the neighbor information of protein atoms in the same class can be partly kept. The one hot representation of each atom type in the atom pairs is concatenated in the same line. The concatenation representation of pairs was written into files line by line. We define the maximum line number as 1,000, which cover almost all of the pair numbers. In order to unify the input format, if the pair number is smaller than 1,000, lines with all 0 will be filled, if the pairs number is larger than 1,000 which is rare, the later part will be removed.

### Network architecture of the model

**Figure 2:**
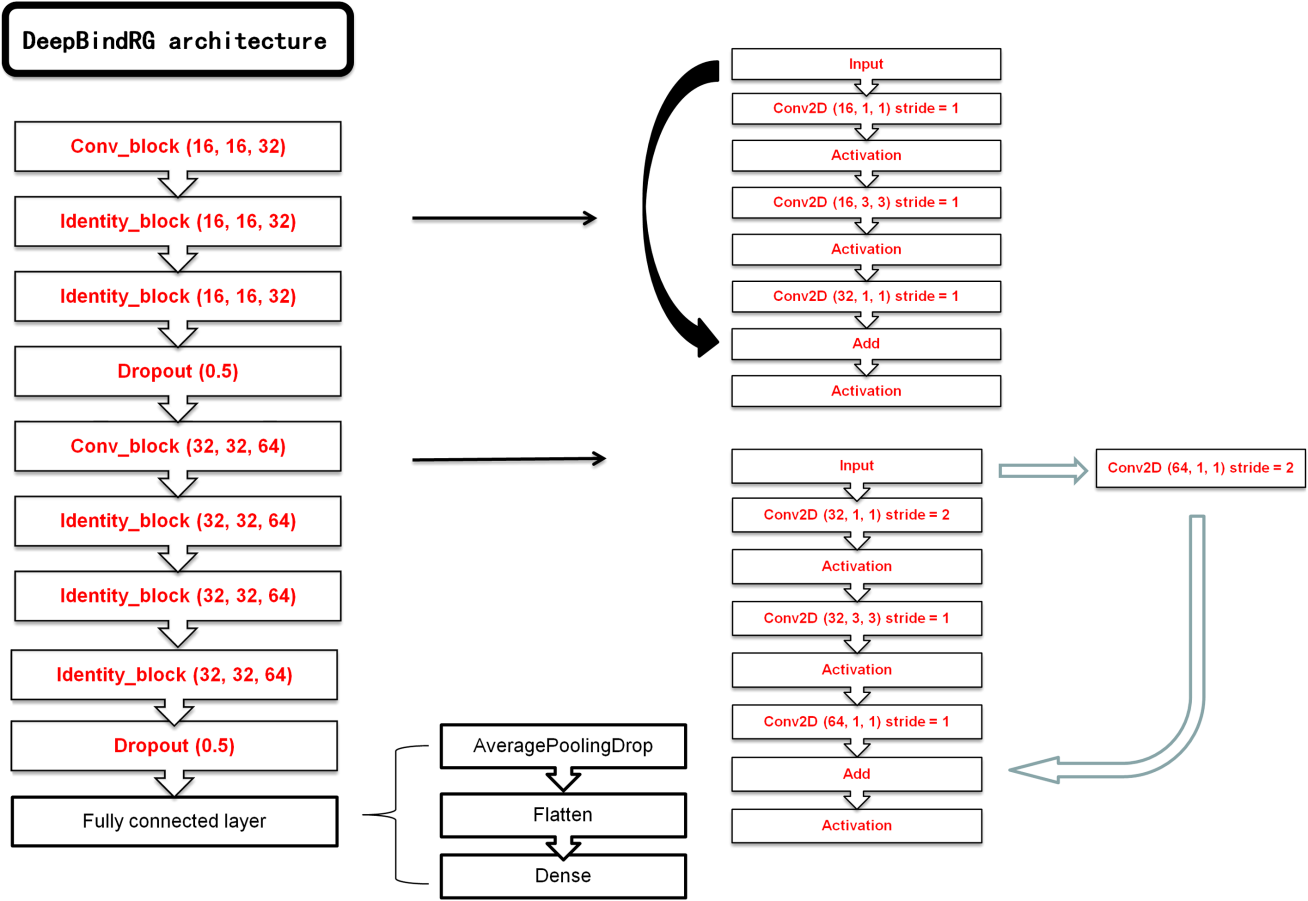
The architecture of our ResNet model.

The keras (Chollet, 2015) package with tensorflow (Abadi et al., 2016) as backend was used to construct the deep neural network model. We have constructed a ResNet and a normal CNN model. The ResNet was chosen as the final network model, and the normal CNN model was used for comparison. The main architecture of ResNet consists of seven blocks, each of which contains one layer with kernel size of 1 × 1, one layer with kernel size of 3 × 3, and one layer with kernel size of 1 × 1. Computational cost was significantly reduced with this type of architecture. At the end of ResNet, a max pool layer and a flatten layer were added to transform 2D feature map to one-dimensional (1D) vector. This 1D vector could be used as input of final dense layer outputting the ultimate prediction. RMSprop optimizer was used to train the network with 0.001 learning rate and 64 examples per mini-batch. Our ResNet model architecture is shown in Fig. 2. The normal CNN model structure is shown in Fig. S1.

### Training and testing

The training process automatically tunes the weights for minimizing the loss function. The independent test set can guide the choice of hyperparameter. We have used different input parameters. For instance, the influence of hydrogen, the influence of atom type, and the influence of atom pair distance, respectively. The hyper-parameters such as epoch, percentage of dropouts, were evaluated. The model complexity influence was evaluated by comparing the performance of normal CNN model and ResNet model. We check the convergences by observing the change in mean squared error (MSE) value both in training and test sets over the increasing epoch number. Figure S2A shows the performance of the final model with different epoch numbers. The optimal performance of validation set is around epoch 20 and the performance has no significant improvement in validation set after this value. More training leads to overfitting and hence we adopt the epoch 20 as the final number.

The limited data with complex network can lead to overfitting. We used different dropout values to check the performance discrepancy among the training and validation sets. Figure S2B shows the performance of the model for the weight dropout of 20–70%. It was found that 50% dropout has the optimal performance; bigger dropouts will reduce the accuracy, while lower dropout cause overfitting. To avoid overfitting, a controlled dropout with multiple iterations is performed and Fig. S2B reveals the 50% dropout corresponding to the lowest MSE value for both the validation and training dataset. The final model with epoch 20, and 50% dropout, is selected for DeepBindRG.

### Evaluation and validation

The metrics such as mean absolute error (MAE), MSE and root mean squared error (RMSE), mean absolute percentage error (MAPE) and symmetric mean absolute percentage error (sMAPE), and correlation coefficient (*R*) value were used to estimate the performance of the deep learning network model. Both MAE and RMSE are most common metrics used to measure the average magnitude of the error. The RMSE gives a relatively high weight to large errors, which is useful when large errors are undesirable. The correlation coefficient *R* was used to measure the strength and direction of a linear relationship between predicted and experimental measured binding affinity. Since the overfitting problem makes the validation extremely important, we choose several independent validations sets to further test the performance, and also compare the performance with the traditional docking score, machine learning score, as well as some recently developed deep learning methods.

### Protein–ligand binding affinity prediction without experimental structure

In order to further validate the performance of DeepBindRG without experimental structures, we choose four datasets randomly from DUD.E database (Mysinger et al., 2012) and obtained affinity data from DUD.E web server. The four datasets are kith, jak2 and egfr, cdk2, which all contain protein structure as well as bunches of active ligands with binding affinity. Since the data from DUD.E have no experimental structure of protein–ligand binding complex, we use autodock vina docking software to generate the protein–ligand binding complex. We used three strategies to choose the conformation which are possibly near native to perform final prediction. They are DeepBindRG_X (the top autodock vina predicted conformations were used as the final prediction), DeepBindRG_Y (all the autodock vina predicted conformations were used as the ligand–protein complex) and DeepBindRG_Z (among all the generated conformation, we selected the top predicted value of DeepBindRG as final prediction). The pocket size was set to include the active binding site, around 25, 25, 25 Å. The docking center is defined as the center of the protein pocket. For each protein–ligand docking, we generate 20 conformations. Each of the conformation is subjected to prediction by the DeepBindRG model, and we choose the top score to represent the protein–ligand binding affinity. We used autodock vina score for comparison with the score of DeepBindRG.

## Results

### DeepBindRG performance on the training, validation, and testing sets

The DeepBindRG model’s performance on the training and test set were shown in Table 1. The correlation coefficient between the prediction scores and experimentally measured binding affinity was assessed with the Pearson’s correlation coefficient (*R*) and standard deviation (RMSE). *R* = 0.6779 is achieved for the training dataset whereas *R* = 0.5829 and 0.5993 for validation and testing datasets. The errors on training and validation sets monitored during deep learning are presented in Fig. S2. Prediction error was measured with RMSE, MAE, MAPE, and sMAPE. In terms of sMAPE, our results are comparable with autodock vina and pafnucy.

**Table 1:** The performance of the ResNet regression model DeepBindRG, Autodock Vina, and Pafnucy.

Data set	*R*	MAE	MSE	RMSE	MAPE	sMAPE	Size
DeepBindRG performance							
Training set	0.6779	1.1153	1.9896	1.4105	21.5282	8.8678	13,500
Validation set	0.5829	1.2067	2.267	1.5057	22.7713	9.6429	1,000
Testing set	0.5993	1.2049	2.241	1.497	22.4016	9.5895	925
CASF-2013	0.6394	1.4829	3.3015	1.817	28.8105	11.9433	195
CSAR_HiQ_NRC_set	0.6585	1.3607	2.9719	1.7239	63.0363	11.1805	343
Astex_diverse_set	0.4657	1.3355	2.6274	1.6209	20.7896	9.9863	74
Autodock Vina performance							
CASF-2013	0.5725	1.9462	5.7647	2.401	38.1536	14.2026	195
CSAR_HiQ_NRC_set	0.5707	1.7268	5.237	2.2884	52.8847	13.89	343
ASTEX_diverse_set	0.422	1.7068	4.8518	2.2027	27.0829	11.7127	74
Pafnucy performance							
CASF-2013	0.5855	1.5131	3.4192	1.8491	30.979	11.784	195
CSAR_HiQ_NRC_set	0.7167	1.2419	2.4787	1.5744	54.5188	9.973	343
Astex_diverse_set	0.5146	1.1732	2.1473	1.4654	19.6549	8.4168	74

The performance of our method after grouping the ligands based on Log*P* reveals the binding affinity of hydrophobic ligands (Group B and C) can be predicted better than the hydrophilic ligands (Group A) which is presented in Table S3. In order to check the robustness of our model, we also performed five random sub-sampling validations (Table S4). It is found that the model has similar performance (*R* = ∼0.6) over each run. The performance of the normal four-layer CNN model on the training and testing set are also shown in Table S5. It is found that our model DeepBindRG performs better than the CNN model (*R* = ∼0.5). We note that the normal CNN have serious overfitting, while adding large dropout would decrease both the testing and training set performance. The performance of using element as atom type is shown in Table S6; the performance (*R* = ∼0.5) is not as good as DeepBindRG, but only using element is more flexible for the application.

### DeepBindRG performance on CASF-2013, CSAR_HiQ_NRC_set, and ASTEX_diverse_set

We have chosen CASF-2013, CSAR_HiQ_NRC_set, and ASTEX_diverse_set as extra testing data set, which contains 195, 343, 74 protein–ligand complexes, respectively. The performance of DeepBindRG on these three extra testing datasets are presented in Table 1, by using *R* value, MAE, MSE, MAPE, sMAPE, and RMSE as performance indicators. The *R* value for CASF-2013 and CSAR_HiQ_NRC_set is about ∼0.6, whereas it is low for Astex_diverse_set (*R* = ∼0.46). It is also observed from Table 1 that pafnucy performs better than DeepBindRG on two datasets out of three, both in terms of correlation coefficient and RMSE. After careful examination, we found there is one case 1YVF of Astex Diverse Set was in the training set of pafnucy. Also, there are 201 cases of the CSAR_HiQ_NRC_set are in the training set of pafnucy, which is about 201/343 overlapping. Also, Pafnucy works well only for native protein–ligand complexes and its performance on docked structure is poor as shown in Table 1. We have compared the performance of autodock vina and pafnucy (Deep learning CNN method) (Stepniewska-Dziubinska et al., 2018) with DeepBindRG, and the results are shown in Table 1. The deep learning method pafnucy achieves *R* value of 0.5855 for CASF-2013, 0.7167 for CSAR_HiQ_NRC_set and 0.5146 for Astex_diverse_set, respectively. The relatively better performance of pafnucy indicates the incorporation of detailed atomic spatial information helps to improve prediction of protein–ligand binding affinity in high resolution crystal structures.

**Figure 3:**
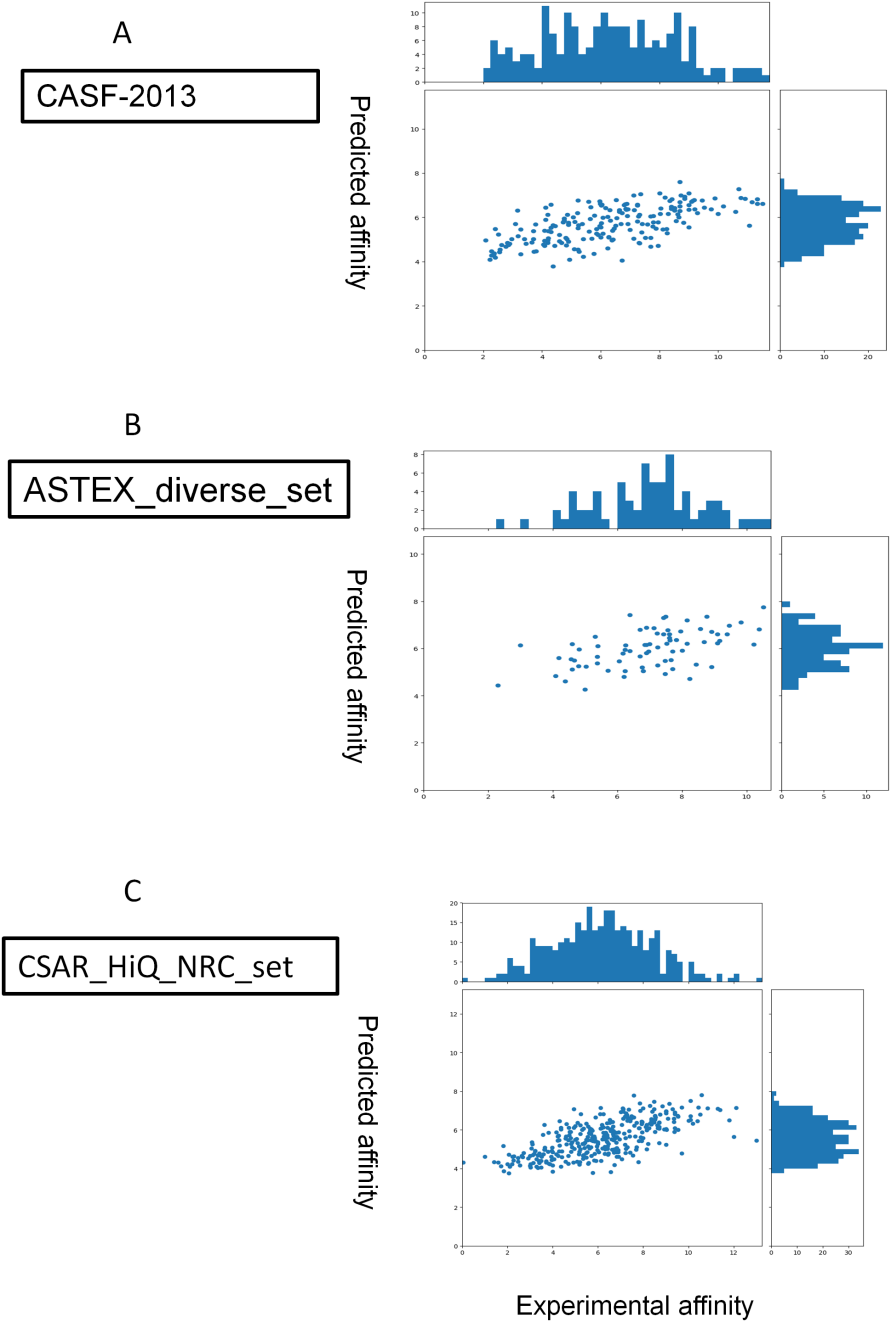
Predictions for three extra validation sets (A, CASF-2013; B, astex_diverse_set; C, CSAR_HiQ_NRC_set).

Figure 3 shows the correlation coefficient between predicted values over the experimental values for the three datasets with only few outliers. We compared the performance of DeepBindRG on the CASF-2013 dataset with other models extracted from the literature reports (Li et al., 2014). The performance of DeepBindRG on the CASF-2013 achieves a *R* value of 0.6394, which is better than other methods such as X-Score, ChemScore, ChemPLP, PLP1, and G-Score, with *R* values of 0.61, 0.59, 0.58, 0.57, and 0.56, respectively. The standard deviation in regression (SD) of DeepBindRG on the CASF-2013 is 1.7306, which is also better than X-Score, ChemScore ChemPLP, PLP1, and G-Score which have SD value of 1.78, 1.82, 1.84, 1.86, and 1.87, respectively. The RMSE value of DeepBindRG on the CASF-2013 is 1.8170, which is higher comparing to the RMSE values of the validation and testing datasets (around 1.5). The possible reason is that many complexes in the CASF-2013 contain relatively small ligands (about 44.62% ligand with size smaller than 40), whereas training dataset has relatively lower percentage of such small ligands (about 32.07% ligand with size smaller than 40).

The performance of DeepBindRG on the CSAR_HiQ_NRC_set achieves an *R* value of 0.6585, and a RMSE value of 1.7239, which indicates the relative strong correlation and small deviation between predicted and experimental measured values. From Fig. 3B, it is observed that the predicted value is highly correlated with the experimental value. Only few outliers such as 1swk and 2c1q have most significant deviation between the experimental affinity value and docking prediction (shown in Fig. S3). The possible reason is that the two connecting aromatic ring regions (marked by green ellipse in figure) occurred in the training data of DeepBindRG model. It should also be noted that the *R* value for the ASTEX_diverse_set is not as good as other datasets (0.422), while it still performs better prediction than the autodock vina.

**Figure 4:**
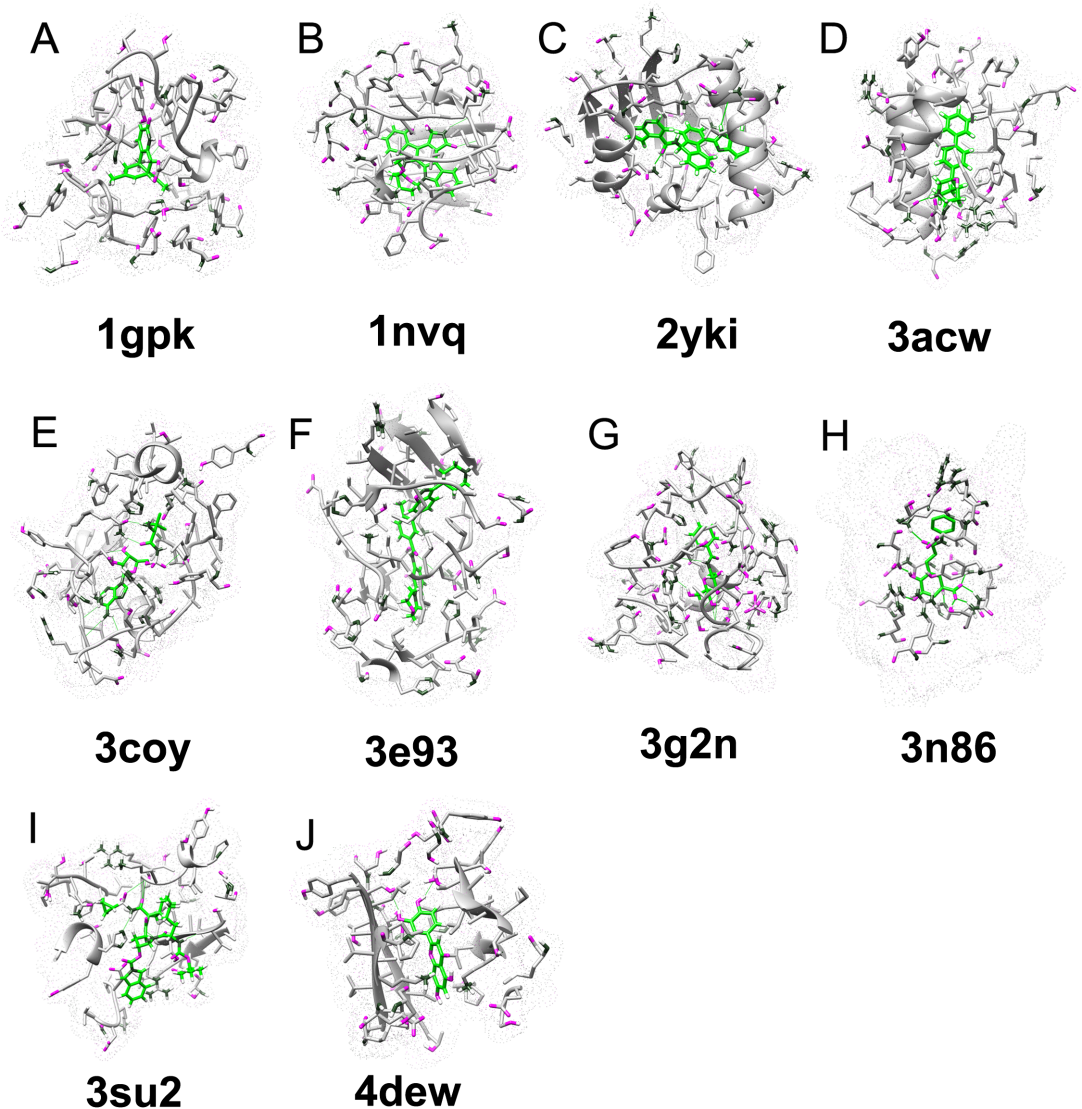
Examples of ligand–protein interaction in the CASF-2013 data set that can be correctly identified by our DeepBindRG, but are not predicted by vina score (DetaG_vina >4, while DetaG_DeepbindRG <2). Among them, the affinity of 4dew is underestimated, while all other nine cases are overestimated. The vina score seems to overestimate pi–pi interaction (A, 1gpk; B, 1nvq; C, 2yki; D, 2acw; F, 3e93) hydrophobic interaction (I, 3su2) and hydrogen bond interaction (E, 3coy; G, 2g2n; H, 3n86), and underestimate polar/electrical interaction, or interaction meditated by water or ion (J, 4edw).

Ten failed predictions by autodock vina on the CASF-2013 were shown in Table 2 and Fig. 4. Six failures are due to overestimation of hydrophobic interaction, especially pi–pi interaction, and three failures are due to overestimation of hydrogen bond interaction. 4edw was seriously underestimated by autodock vina, because of the surrounding charged amino acids. The interaction mediated by water or ion may be seriously underestimated by autodock vina, as in the 4edw case, the pocket is formed in the core of protein, it can contain more water molecules than other polar cases. The proteins like 1nvq, 2yki, 3coy, 3e93, 3g2n, and 4dew have extra volume space after ligand binding, indicate possible solvent effect on these cases (Table S7). We suspect that underestimation of the water effect will artificially increase the autodock vina predicted binding affinity of hydrophobic dominant binding (e.g. 1nvq, 2yki, 3coy, 3e93), while decrease in autodock vina predicted affinity is due to polar dominant binding (4dew). The autodock vina predicted binding affinity of 3g2n is overestimated due to hydrogen bond and charge–charge interaction.

**Table 2:** The selected cases that DeepBindRG had significant better performance than the vina score in the CASF-2013 data set.

PDBID	Experimental affinity	Vina score	DeltaG_vina	DeepBindRG predicted affinity	DeltaG_DeepbindRG
2yki	9.46	16.1137	6.6537	8.1597	1.3003
4dew	7	0.6853	6.3147	5.8712	1.1288
3acw	4.76	10.2398	5.4798	6.2444	1.4844
3n86	5.64	10.9262	5.2862	6.1666	0.5266
1gpk	5.37	10.1323	4.7623	6.3859	1.0159
3e93	8.85	13.3378	4.4878	7.3438	1.5062
3g2n	4.09	8.5575	4.4675	4.9792	0.8892
3su2	7.35	11.6916	4.3416	6.9883	0.3617
1nvq	8.25	12.5577	4.3077	6.6401	1.6099
3coy	6.02	10.2338	4.2138	5.9635	0.0565
Vina MAPE		79.9297			
Vina sMAPE		15.3444			
Vina correlation		0.4362			
DeepBindRG MAPE		29.3784			
DeepBindRG sMAPE		7.5111			
DeepBindRG correlation		0.8519			

**Note:**

We define the significant better as DeltaG_vina >4, while DeltaG_DeepbindRG <2. The average error and correlation coefficient are provided below the table.

### DeepBindRG performance on DUD.E dataset

In order to further test the effectiveness of our method, we selected the data samples from DUD.E dataset. Since the DUD.E dataset does not contain the experimental protein–ligand binding complex, it is a much more challenging test for our DeepBindRG model. The performance of DeepBindRG, Autodock vina, and pafnucy on four randomly selected subsets are shown in Table 3. The autodock vina score has larger RMSE values for all the four subsets, 3.9817, 2.4542, 2.5514, and 1.795, which indicates its prediction hardness in some of such challenge cases. We have tested the performance of our model with three strategies for final conformation selection, X, Y, and Z.

**Table 3:** The performance of the DeepBindRG and autodock vina on the datasets from DUD.E database.

	*R*	MAE	MSE	RMSE	Size
kith dataset					
DeepBindRG_X*	0.4742	1.823	4.2923	2.0718	57
DeepBindRG_Y*	0.3156	1.3382	2.6312	1.6221	1,127
DeepBindRG_Z*	0.5588	2.123	5.336	2.31	57
Vina score	0.6664	3.8567	15.8536	3.9817	57
Pafnucy*	0.4673	3.2789	11.6922	3.4194	57
Jak2 dataset					
DeepBindRG_X*	−0.028	1.1715	2.2772	1.509	107
DeepBindRG_Y*	0.0189	1.4913	3.2848	1.8124	2,078
DeepBindRG_Z*	−0.0195	0.9314	1.525	1.2349	107
Vina score	0.1037	2.1678	6.0232	2.4542	107
Pafnucy*	−0.1186	1.0141	1.5354	1.2391	107
Egfr dataset					
DeepBindRG_X*	−0.0705	1.124	2.1048	1.4508	542
DeepBindRG_Y*	−0.0241	1.3153	2.8598	1.6911	10,614
DeepBindRG_Z*	−0.0314	1.043	1.7365	1.3177	542
Vina score	0.0146	2.2055	6.5095	2.5514	542
Pafnucy*	0.1701	1.1253	1.8209	1.3494	542
Cdk2 dataset					
DeepBindRG_X*	0.2205	1.0317	1.61	1.2689	474
DeepBindRG_Y*	0.1947	1.3589	2.5988	1.6121	9,027
DeepBindRG_Z*	0.2797	0.7854	0.9238	0.9612	474
Vina score	0.0554	1.5393	3.2222	1.795	474
Pafnucy*	0.1230	0.7346	0.8277	0.9098	474

**Notes:**

DeepBindRG_X*: the top autodock vina predicted conformations were used as the final prediction.

DeepBindRG_Y*: all the autodock vina predicted conformations were used as the ligand–protein complex.

DeepBindRG_Z*: among all the generated conformation, we selected the top predicted value of DeepBindRG as final prediction.

Pafnucy*: among all the generated conformation, we selected the top predicted value of Pafnucy as final prediction.

Except kith dataset, the DeepBindRG_Z shown better performance than DeepBindRG_X, DeepBindRG_Y in terms of RMSE. The correlation coefficient between predicted and experimental binding affinities are presented in Fig. S4. The DeepBindRG_Z resulted in top predicted score of DeepBindRG as final prediction among all the generated conformation has better performance than other strategy. Although, the most predictive model seems to be DeepBindRG_Z on the Kith dataset (*R* = 0.66), But it is the one with the largest RMSE. All other models are completely unpredictive and the RMSE is just quantifying the amplitude of the noise or the spread of experimental values themselves. We notice that for some prediction cases of DeepBindRG, the *R* value is close to zero, while the RMSE value is relatively small. However, it should be noted that the accuracy of DeepBindRG has a lot of room for further improvement. The major challenges are: (1) how to generate conformations as close as possible to native structure, and (2) how to select a conformation that is native-like. From Table 3, it is observed that the inconsistent prediction of all the three methods on four datasets with no experimental structure indicates the discrepancy between testing and real application. The predictions of Jak2 and egfr datasets are extremely poor, this is because of high flexibility of amino acid residues in the ligand binding pocket. Since the ligand binding pocket is flexible, receptor reshapes around pockets, and stabilizes the complex by complementary hydrophobic interactions and specific hydrogen bonds with the ligand. The fluctuating nature of ligand binding pockets and inaccurate identification of near native pockets may be the reason for inconsistent prediction of all the three methods on DUD.E datasets.

## Discussion

The increasing availability of experimental protein–ligand complexes have allowed us to learn the underlying rules of protein–ligand interactions from the data by deep learning method. However, our work shows several challenges need to overcome before deep learning-based protein–ligand affinity estimators when applied to real applications. Both the DeepBindRG and pafnucy have poor performance on the four datasets from DUD.E while comparing with other extra test sets which have experimental conformation, this indicates the current method needs improvement in close-to-real application. The structure-based method requires high accurate protein–ligand binding conformation before prediction, while the accurate protein–ligand binding conformation is hard to obtain and such process is relatively time-consuming.

There are several situations that can seriously affect the prediction accuracy: (1) the native-like conformation is not in the conformation pool (usually generated by molecular docking); (2) the criterion to select native-like conformation is not accurate enough; (3) the model trained by the accurate experimental structures cannot identify near to native conformation. The distribution discrepancy between training data and real application data is another challenge. In the training dataset, the strong binders are dominant, while in the real application, the non-binders are dominant, and weak binders are usually more than the strong binders. This can lead to the poor performance of the model. Our work shows above generalized problem of current deep learning methods, and indicates protein–ligand binding estimator models should focus to solve such problem instead of pursuing high accuracy on data which have experimental structure. A possible solution is to add many near native conformations to the training data set, for instance, the near-native as positive, and docked non-binder complexes as negative. Another possible solution is to increase the accuracy and efficiency of the docking method in sampling native-like conformation.

## Conclusion

In the present work, we developed a deep learning model “DeepBindRG” for identifying native-like protein–ligand complex. The accuracy of our method for evaluating protein–ligand binding affinity is comparable with pafnucy which uses much complicated 4D input representation. In normal cases, the simple deep learning model is susceptible to the artificial enrichment of the dataset, resulting in overly optimistic predictions of training dataset and test data set; however, DeepBindRG has performed well for several external independent data sets from different source. Since the datasets from DUD.E do not contain a native protein–ligand, this test is very challenging and close to real application. In this paper, we demonstrated the potential of ResNet and CNNs in identifying native-like protein–ligand complexes than other publicly available popular methods. Our result shows the more complicated CNN model ResNet can improve the prediction result comparing to the normal CNN. We also show that our model using more elaborate atom types from force field as input performs better than the simple element-based input. Using more spatial information of the interface between protein and ligand will aid to predict the affinity strength by implicit learning critical factors that determine protein–ligand interactions. By comparing with the 4D based CNN model pafnucy, our research shows the generalized problem (extreme dependent on native protein–ligand conformation) of the current deep learning model in protein–ligand affinity prediction, and indicates several critical point for developing high accurate protein–ligand affinity model: keeping spatial information; using deeper neural network to learn more abstract information; making the training and testing data set have the same feature distribution as the real application; keeping the elaborately atom type information. We believe DeepBindRG is a promising model to facilitate the drug development process, especially in discovering novel biologically active lead compounds for specific therapeutic protein targets. Our software is freely available for download in the GitHub public repository (https://github.com/haiping1010/DeepBindRG).

## Supplemental Information

10.7717/peerj.7362/supp-1 Supplemental Information 1 The range of experimental binding affinity. The range of binding affinity for training, test, validation set and three extra test test were given in the table.

10.7717/peerj.7362/supp-2 Supplemental Information 2 The atom type used in the present work. The atom types of ligand and protein used in our input data of DeepBindRG model were given.

10.7717/peerj.7362/supp-3 Supplemental Information 3 Number and percentage of ligands in the dataset grouped based on logP. The group A have logP<-1, group B have -1<=loP <1 and group C have logP>=1 respectively.

10.7717/peerj.7362/supp-4 Supplemental Information 4 Performance of DeepBindRG after grouping the ligands based on LogP. The group A have logP<-1, group B have -1<=loP <1 and group C have logP>=1, respectively.

10.7717/peerj.7362/supp-5 Supplemental Information 5 The performance of random sub-sampling validation. Five random sub-sampling validation have been taken, and the results are given in the table.

10.7717/peerj.7362/supp-6 Supplemental Information 6 The performance of normal CNN model. The overfitting problem is difficult to solve, because as we continue to increase the dropout percentage, the accuracy for the test set and validation set will deceases as well. The overfitting problem is difficult to solve, because as we continue to increase the dropout percentage, the accuracy for the test set and validation set will deceases as well.

10.7717/peerj.7362/supp-7 Supplemental Information 7 The performance of the DeepBindRG_ele model which uses element as atom type. The performance of same model arctecture on the simplified input representation which uses element as atom type are not as good as DeepBindRG.

10.7717/peerj.7362/supp-8 Supplemental Information 8 The cavity volume of the 10 cases is shown in the Figure 4. It was calculated by Channel Finder program in 3v website with outer probe radius 10 and inner probe radius. It was calculated by Channel Finder program in 3v website with outer probe radius 10 and inner probe radius.

10.7717/peerj.7362/supp-9 Supplemental Information 9 The architecture of the CNN model. The normal CNN model was used as a comparision. The detailed information of each layers was given.

10.7717/peerj.7362/supp-10 Supplemental Information 10 The optimal epoch number and optimal dropout percentage were systematically determined. The performance of different epoch number was shown in Panel A, showing the optimal epoch number is around 20. The performance of different dropout percentage for epoch 20 was shown in Panel B, and the optimal dropout percentage is around 50%.

10.7717/peerj.7362/supp-11 Supplemental Information 11 Two most significant outliers for the prediction of CSAR_HiQ_NRC_set dataset. The possible reason is that the two connect aromatic ring regions (marked by green ellipse) rarely occurred in the training data of DeepBindRG model.

10.7717/peerj.7362/supp-12 Supplemental Information 12 Correlation coefficient between predicted and experimental binding affinity for the DUD.E datasets. The results of DeepBindRG_Z and Pafnucy on 4 datasets from DUD.E database. The left panel is the DeepBindRG_Z, the right panel is the Pafnucy.

## Additional Information and Declarations

### Data Availability

The following information was supplied regarding data availability: Raw data and code are available at GitHub: https://github.com/haiping1010/DeepBindRG.
