# Supplementary material for: DeepBindRG: a deep learning based method for estimating effective protein–ligand affinity
Source: PeerJ. 2019 Jul 25;7:e7362. doi: 10.7717/peerj.7362 (PMC6661145; doi:10.7717/peerj.7362)
Supplement: Supplemental Information 1 — The range of binding affinity for training, test, validation set and three extra test test were given in the table. [file peerj-07-7362-s001.docx]

**Supplementary Table S1(A).** The range of experimental binding affinity

| Data set | Affinity range of group1 | Affinity range of group2 | Affinity range of group3 |
| --- | --- | --- | --- |
| training | 0.82~13 | 0.4~12.33 | 0.45~15.22 |
| validation | 0.96~11.32 | 1.26~10.82 | 2.06~14.39 |
| test | 1.7~9.41 | 1.24~9.92 | 1.44~13 |
| Astex_diverse_set | 4.57~7.7 | 2.3~8.57 | 3~10.52 |
| CSAR_HiQ_NRC_set | 1~9.4 | 0.05~13 | 1.4~12.1 |
| Core_set | 2.23~11.06 | 2.27~10.6 | 2.07~11.52 |
