# Supplementary material for: DeepBindRG: a deep learning based method for estimating effective protein–ligand affinity
Source: PeerJ. 2019 Jul 25;7:e7362. doi: 10.7717/peerj.7362 (PMC6661145; doi:10.7717/peerj.7362)
Supplement: Supplemental Information 2 — The atom types of ligand and protein used in our input data of DeepBindRG model were given. [file peerj-07-7362-s002.docx]

**Supplementary Table S1(B).** The atom type used in the present work.

| **Model** | **Atom type of ligand** | **Atom type of protein** | **Total dimension for ligand-protein pair** |
| --- | --- | --- | --- |
| DeepBindRG | "c","cs","c1","c2","c3","ca","cp","cq","cc","cd","ce","cf","cg","ch","cx","cy","cu","cv","cz","h1","h2","h3","h4","h5","ha","hc","hn","ho","hp","hs","hw","hx","f","cl","br","i","n","n1","n2","n3","n4","na","nb","nc","nd","ne","nf","nh","no","ns","nt","nx","ny","nz","n+","nu","nv","n7","n8","n9","o","oh","os","ow","p2","p3","p4","p5","pb","pc","pd","pe","pf","px","py","s","s2","s4","s6","sh","ss","sx","sy","B" | 'C','CA','CB','CC','CK','CM','CN','CQ','CR','CT','CV','CW','C*','F','H','HC','H1','H2','H3','HA','H4','H5','HO','HS','HW','HP','N','NA','NB','NC','N2','N3','N*','O','OW','OH','OS','O2','P','S','SH' | 125 |
| Element only model | "c","h","f","cl","br","i","n","o","p","s","B" | 'C','F','H','N','O','P','S' | 18 |
