# Supplementary material for: DeepBindRG: a deep learning based method for estimating effective protein–ligand affinity
Source: PeerJ. 2019 Jul 25;7:e7362. doi: 10.7717/peerj.7362 (PMC6661145; doi:10.7717/peerj.7362)
Supplement: Supplemental Information 3 — The group A have logP<-1, group B have -1<=loP <1 and group C have logP>=1 respectively. [file peerj-07-7362-s003.docx]

**Supplementary Table S2.** Number and percentage of ligands in the dataset grouped based on logP. (The group A have logP<-1, group B have -1<=loP <1 and group C have logP>=1 respectively)

| Dataset | Number of group A | Number of group B | Number of group C | Percentage of group A | Percentage of group B | Percentage of group C |
| --- | --- | --- | --- | --- | --- | --- |
| training | 1846 | 2249 | 9405 | 0.1367 | 0.1666 | 0.6967 |
| validation | 156 | 164 | 680 | 0.156 | 0.164 | 0.68 |
| test | 123 | 137 | 665 | 0.1330 | 0.1481 | 0.7189 |
| astex_diverse_set | 4 | 10 | 60 | 0.0541 | 0.1351 | 0.8108 |
| core_set | 47 | 32 | 116 | 0.241 | 0.1641 | 0.5949 |
| CSAR_HiQ | 96 | 66 | 181 | 0.2799 | 0.1924 | 0.5277 |
