# Supplementary material for: DeepBindRG: a deep learning based method for estimating effective protein–ligand affinity
Source: PeerJ. 2019 Jul 25;7:e7362. doi: 10.7717/peerj.7362 (PMC6661145; doi:10.7717/peerj.7362)
Supplement: Supplemental Information 4 — The group A have logP<-1, group B have -1<=loP <1 and group C have logP>=1, respectively. [file peerj-07-7362-s004.docx]

**Supplementary Table S3.** Performance of DeepBindRG after grouping the ligands based on LogP. (The group A have logP<-1, group B have -1<=loP <1 and group C have logP>=1 respectively)

| Dataset Name | Class | R | MAE | MSE | RMSE | MAPE | sMAPE |
| --- | --- | --- | --- | --- | --- | --- | --- |
| Training set | Group A | 0.5356 | 1.2024 | 2.3183 | 1.5226 | 29.1896 | 11.0883 |
|  | Group B | 0.5544 | 1.2178 | 2.2923 | 1.514 | 28.0323 | 10.8664 |
|  | Group C | 0.6666 | 1.0738 | 1.8528 | 1.3612 | 18.4691 | 8.1066 |
|  | | | | | | | |
| Test set | Group A | 0.3146 | 1.3079 | 2.4914 | 1.5784 | 29.697 | 12.0051 |
|  | Group B | 0.3306 | 1.3141 | 2.6458 | 1.6266 | 31.9007 | 12.0847 |
|  | Group C | 0.5658 | 1.1576 | 2.1241 | 1.4574 | 18.9806 | 8.7162 |
|  |  |  |  |  |  |  |  |
| Validation set | Group A | 0.4277 | 1.2404 | 2.2615 | 1.5038 | 27.4044 | 11.2924 |
|  | Group B | 0.4275 | 1.2653 | 2.3746 | 1.541 | 29.6903 | 11.4607 |
|  | Group C | 0.5795 | 1.1859 | 2.2097 | 1.4865 | 19.9747 | 9.0037 |
|  |  |  |  |  |  |  |  |
| astex_diverse_set | Group A | -0.4787 | 1.5382 | 2.5596 | 1.5999 | 24.9154 | 13.0519 |
|  | Group B | 0.6532 | 1.4539 | 2.5941 | 1.6106 | 29.8849 | 12.5465 |
|  | Group C | 0.3957 | 1.3023 | 2.6375 | 1.624 | 18.9986 | 9.4525 |
|  |  |  |  |  |  |  |  |
| CSAR_HiQ | Group A | 0.3987 | 1.2669 | 2.3481 | 1.5323 | 36.7059 | 12.519 |
|  | Group B | 0.4053 | 1.4538 | 3.9973 | 1.9993 | 209.7753 | 13.0725 |
|  | Group C | 0.6473 | 1.3765 | 2.9289 | 1.7114 | 23.4945 | 10.0913 |
|  |  |  |  |  |  |  |  |
| core_set | Group A | 0.4169 | 1.4658 | 3.1588 | 1.7773 | 35.4467 | 13.7631 |
|  | Group B | 0.5145 | 1.6984 | 3.9174 | 1.9792 | 39.3044 | 14.606 |
|  | Group C | 0.6484 | 1.4303 | 3.1894 | 1.7859 | 23.2268 | 10.7151 |
