# Supplementary material for: DeepBindRG: a deep learning based method for estimating effective protein–ligand affinity
Source: PeerJ. 2019 Jul 25;7:e7362. doi: 10.7717/peerj.7362 (PMC6661145; doi:10.7717/peerj.7362)
Supplement: Supplemental Information 5 — Five random sub-sampling validation have been taken, and the results are given in the table. [file peerj-07-7362-s005.docx]

**Supplementary Table S4.** The performance of random sub-sampling validation.

| **Cross validation ID** | **Training set（13500）** | | | | **Validation set（1000）** | | | | **Testing set（925）** | | | |
| --- | --- | --- | --- | --- | --- | --- | --- | --- | --- | --- | --- | --- |
|  | **R value** | **MAE** | **MSE** | **RMSE** | **R value** | **MAE** | **MSE** | **RMSE** | **R value** | **MAE** | **MSE** | **RMSE** |
| **1** | **0.6709** | **1.1179** | **1.9880** | **1.4100** | **0.5547** | **1.2476** | **2.5093** | **1.5841** | **0.6086** | **1.2130** | **2.3011** | **1.5169** |
| **2** | **0.6833** | **1.2961** | **2.6046** | **1.6139** | **0.5855** | **1.3887** | **2.9603** | **1.7205** | **0.5654** | **1.3950** | **3.0486** | **1.7460** |
| **3** | **0.6781** | **1.1226** | **2.0105** | **1.4179** | **0.5392** | **1.2459** | **2.4329** | **1.5598** | **0.5625** | **1.2392** | **2.4137** | **1.5536** |
| **4** | **0.6660** | **1.1592** | **2.1535** | **1.4675** | **0.6021** | **1.2740** | **2.5811** | **1.6066** | **0.6008** | **1.2923** | **2.6219** | **1.6192** |
| **5** | **0.6802** | **1.1265** | **1.9990** | **1.4139** | **0.5827** | **1.2774** | **2.6109** | **1.6158** | **0.5334** | **1.2146** | **2.4601** | **1.5685** |
| **Average** | **0.6757** | **1.1645** | **2.1511** | **1.4646** | **0.5729** | **1.2867** | **2.6189** | **1.6174** | **0.5742** | **1.2708** | **2.5691** | **1.6009** |
