# Supplementary material for: DeepBindRG: a deep learning based method for estimating effective protein–ligand affinity
Source: PeerJ. 2019 Jul 25;7:e7362. doi: 10.7717/peerj.7362 (PMC6661145; doi:10.7717/peerj.7362)
Supplement: Supplemental Information 6 — The overfitting problem is difficult to solve, because as we continue to increase the dropout percentage, the accuracy for the test set and validation set will deceases as well. [file peerj-07-7362-s006.docx]

**Supplementary Table S5.** The performance of normal CNN model. The overfitting problem is difficult to solve, because as we continue to increase the dropout percentage, the accuracy for the test set and validation set will deceases as well.

| Metrics | Training set(135000) | Validation set(1000) | Test set(925) |
| --- | --- | --- | --- |
| R value | 0.9849 | 0.5057 | 0.543 |
| MAE | 0.2569 | 1.3069 | 1.263 |
| MSE | 0.1087 | 2.6994 | 2.5302 |
| RMSE | 0.3298 | 1.643 | 1.5907 |
