# Supplementary material for: DeepBindRG: a deep learning based method for estimating effective protein–ligand affinity
Source: PeerJ. 2019 Jul 25;7:e7362. doi: 10.7717/peerj.7362 (PMC6661145; doi:10.7717/peerj.7362)
Supplement: Supplemental Information 7 — The performance of same model arctecture on the simplified input representation which uses element as atom type are not as good as DeepBindRG. [file peerj-07-7362-s007.docx]

**Supplementary Table S6.** The performance of the DeepBindRG_ele model which uses element as atom type.

| Metrics | Training set(135000) | Test set(1000) | Validation set(925) |
| --- | --- | --- | --- |
| R value | 0.5715 | 0.5350 | 0.5422 |
| MAE | 1.2788 | 1.2786 | 1.2973 |
| MSE | 2.5463 | 2.5389 | 2.5768 |
| RMSE | 1.5957 | 1.5934 | 1.6052 |
