# Supplementary material for: DeepBindRG: a deep learning based method for estimating effective protein–ligand affinity
Source: PeerJ. 2019 Jul 25;7:e7362. doi: 10.7717/peerj.7362 (PMC6661145; doi:10.7717/peerj.7362)
Supplement: Supplemental Information 8 — It was calculated by Channel Finder program in 3v website with outer probe radius 10 and inner probe radius. [file peerj-07-7362-s008.docx]

**Supplementary Table S7.** The cavity volume of the 10 cases is shown in the Figure 4. It was calculated by Channel Finder program in 3v website with outer probe radius 10 and inner probe radius

| PDBID | Pocket volume(Å3) | Pocket volume minus ligand volume(Å3) |
| --- | --- | --- |
| 1gpk | 374 | 48 |
| 1nvq | 609 | 196 |
| 2yki | 831 | 521 |
| 3acw | 327 | 96 |
| 3coy | 636 | 349 |
| 3e93 | 942 | 448 |
| 3g2n | 565 | 176 |
| 3n86 | 170 | 31 |
| 3su2 | 192 | 34 |
| 4dew | 356 | 149 |
