# Supplementary figures and images for: DeepBindRG: a deep learning based method for estimating effective protein–ligand affinity
Source: PeerJ. 2019 Jul 25;7:e7362. doi: 10.7717/peerj.7362 (PMC6661145; doi:10.7717/peerj.7362)

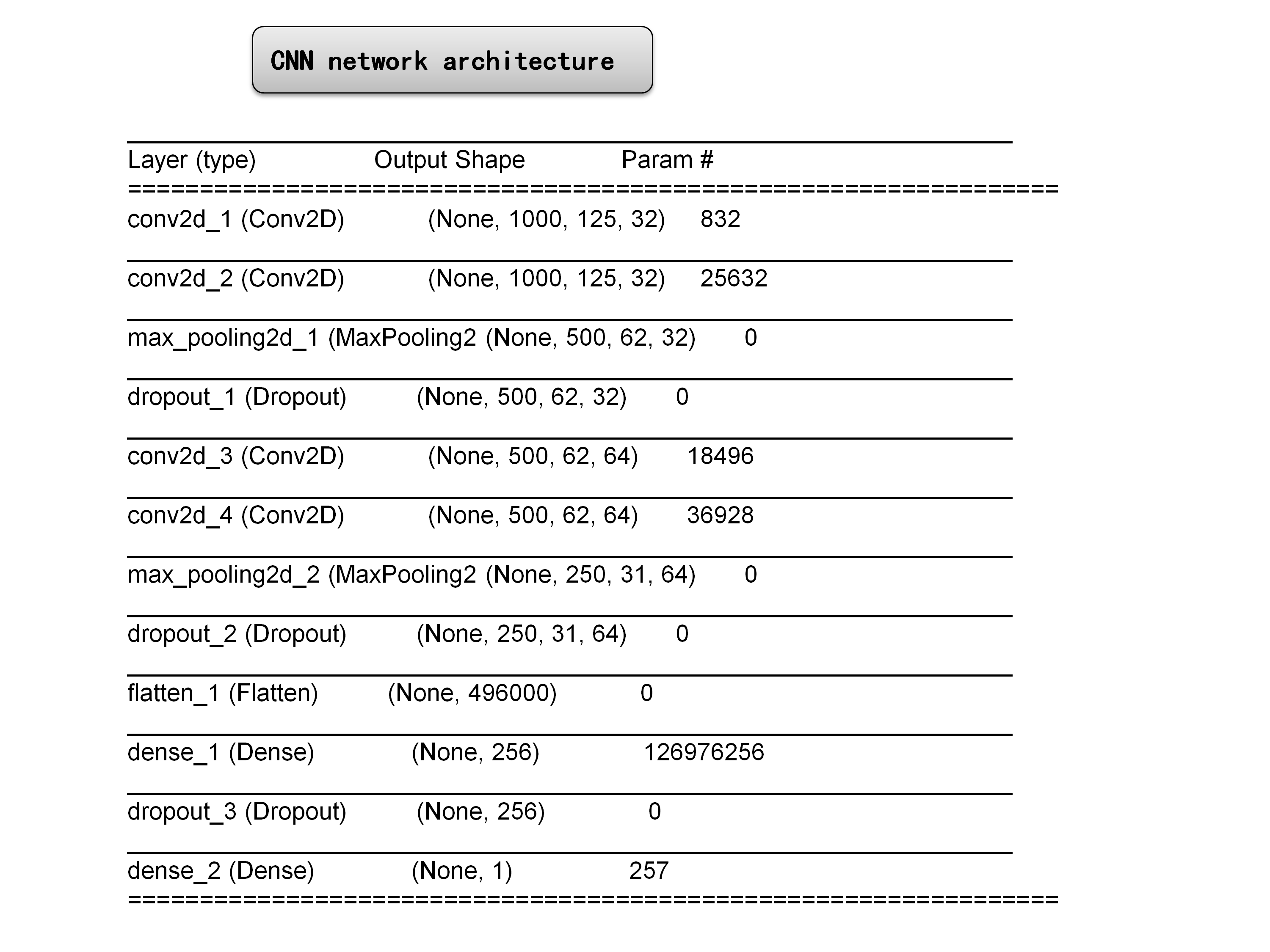

Supplement: Supplemental Information 9 — The normal CNN model was used as a comparision. The detailed information of each layers was given. [file peerj-07-7362-s009.png]

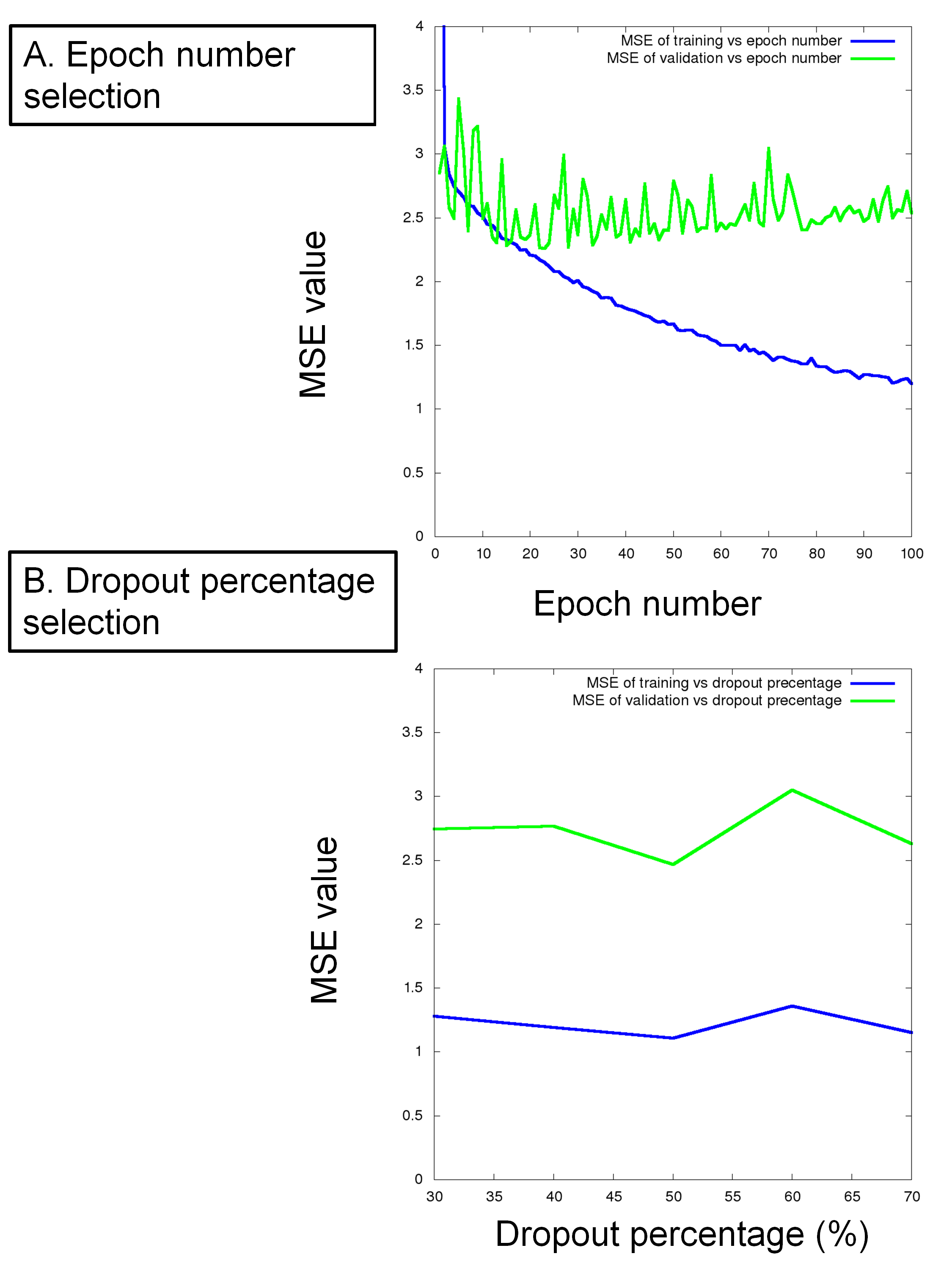

Supplement: Supplemental Information 10 — The performance of different epoch number was shown in Panel A, showing the optimal epoch number is around 20. The performance of different dropout percentage for epoch 20 was shown in Panel B, and the optimal dropout percentage is around 50%. [file peerj-07-7362-s010.png]

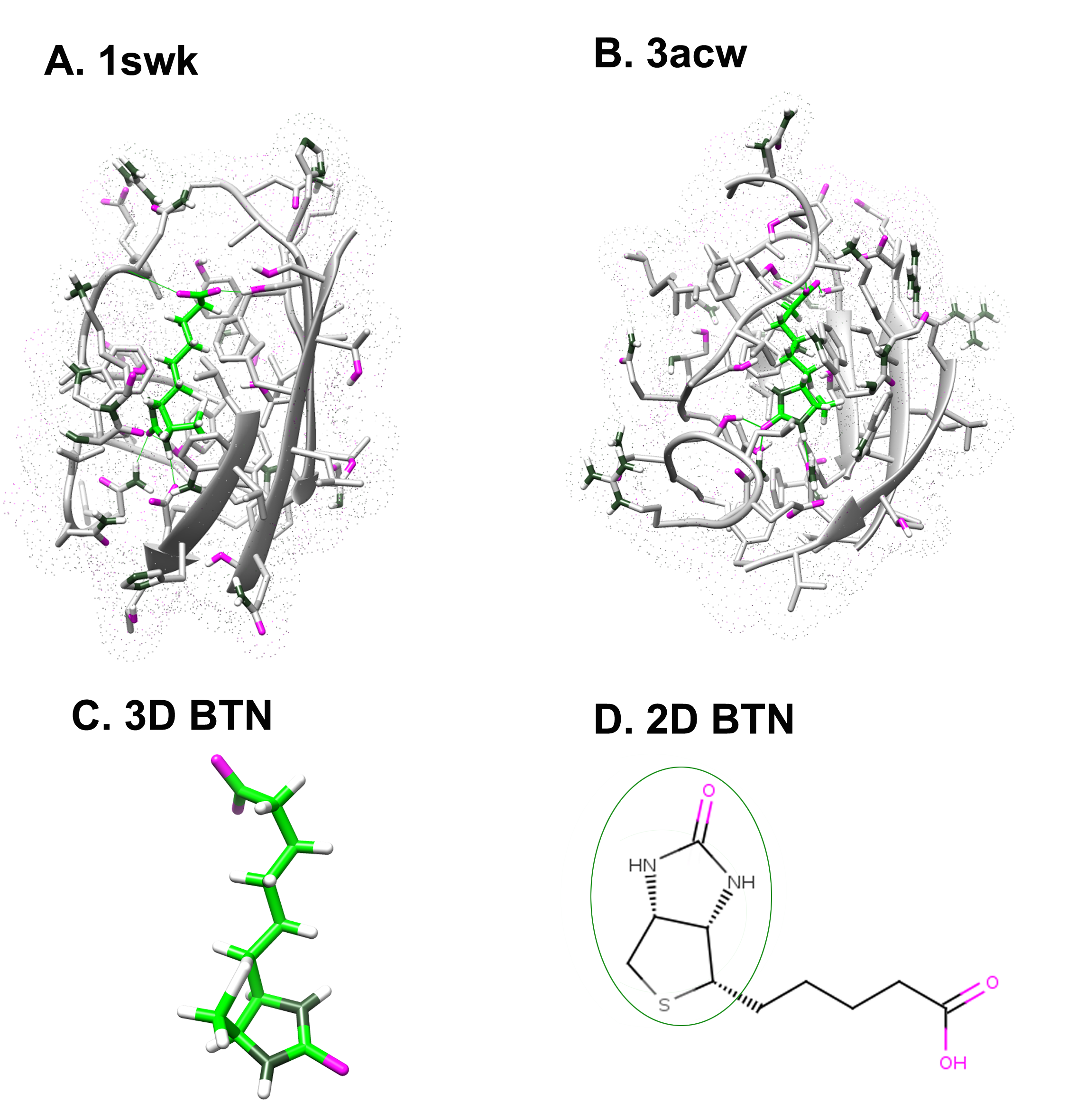

Supplement: Supplemental Information 11 — The possible reason is that the two connect aromatic ring regions (marked by green ellipse) rarely occurred in the training data of DeepBindRG model. [file peerj-07-7362-s011.png]

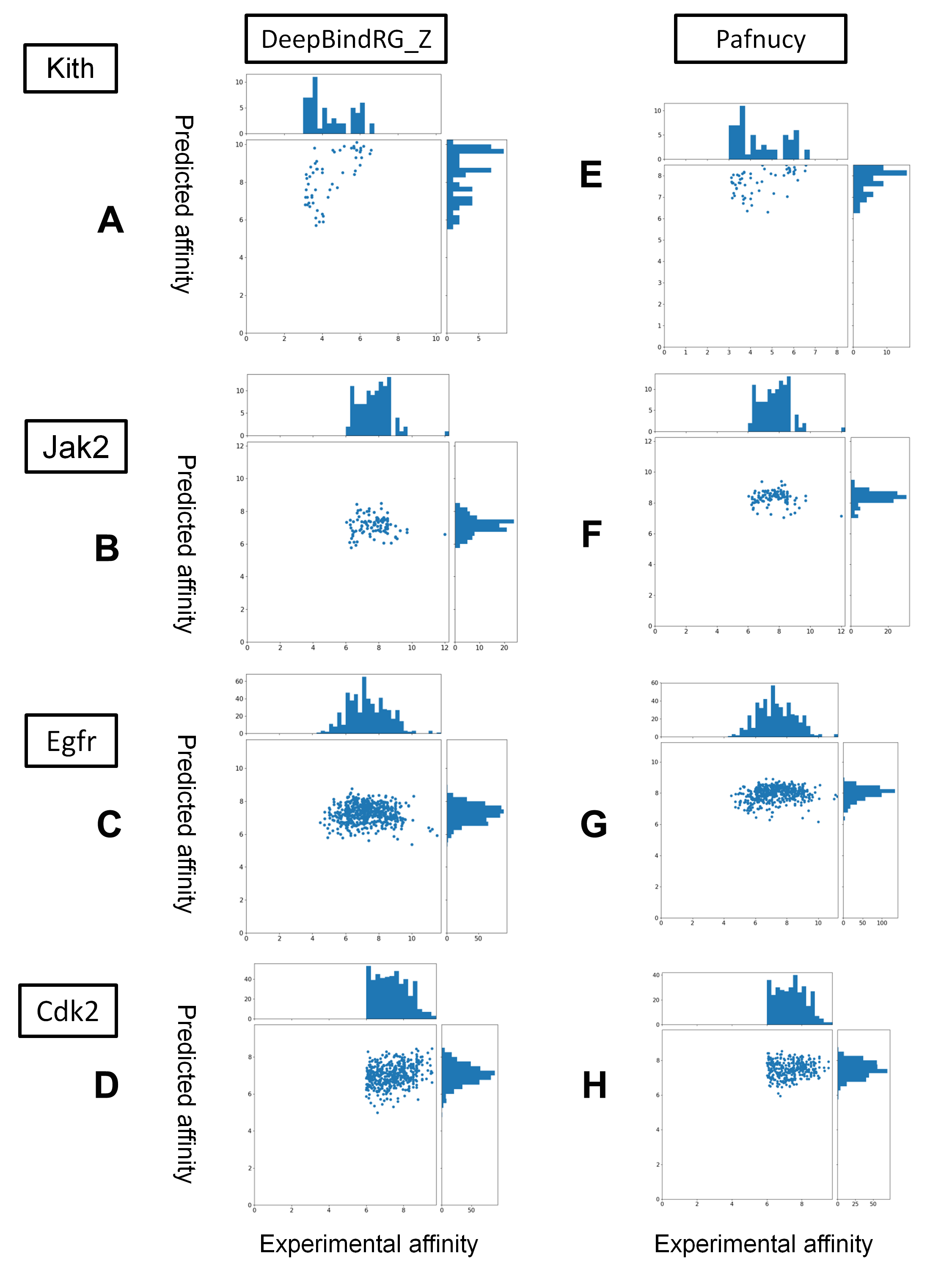

Supplement: Supplemental Information 12 — The results of DeepBindRG_Z and Pafnucy on 4 datasets from DUD.E database. The left panel is the DeepBindRG_Z, the right panel is the Pafnucy. [file peerj-07-7362-s012.png]
